# Task-specific training versus usual care to improve upper limb function after stroke: the “Task-AT Home” randomised controlled trial protocol

**DOI:** 10.3389/fneur.2023.1140017

**Published:** 2023-06-29

**Authors:** Paulette van Vliet, Leeanne Mary Carey, Ailie Turton, Gert Kwakkel, Kerrin Palazzi, Christopher Oldmeadow, Andrew Searles, Heidi Lavis, Sandy Middleton, Margaret Galloway, Bleydy Dimech-Betancourt, Sophie O'Keefe, Meredith Tavener

**Affiliations:** ^1^School of Health Sciences, College of Health, Medicine and Wellbeing, The University of Newcastle, Callaghan, NSW, Australia; ^2^Occupational Therapy, School of Allied Health, Human Services and Sport, La Trobe University, Melbourne, VIC, Australia; ^3^Brain Research Institute, Florey Institute of Neuroscience and Mental Health, Faculty of Medicine, Dentistry and Health Sciences, University of Melbourne, Melbourne, VIC, Australia; ^4^School of Health and Social Wellbeing, Faculty of Health and Applied Sciences, University of the West of England, Bristol, England, United Kingdom; ^5^Department of Rehabilitation Medicine, Amsterdam Movement Science and Amsterdam Neuroscience, Amsterdam University Medical Center, Amsterdam, Netherlands; ^6^Clinical Research Design, Information Technology and Statistical Support (CReDITSS) Unit, Hunter Medical Research Institute, New Lambton Heights, NSW, Australia; ^7^Hunter Medical Research Institute, The University of Newcastle, New Lambton, NSW, Australia; ^8^School of Medicine and Public Health, College of Health, Medicine and Wellbeing, The University of Newcastle, Callaghan, NSW, Australia; ^9^Nursing Research Institute, St Vincent’s Network Sydney and Australian Catholic University, Sydney, NSW, Australia

**Keywords:** stroke, upper limb, therapy, rehabilitation, task-specific

## Abstract

**Background:**

Sixty percent of people have non-functional arms 6 months after stroke. More effective treatments are needed. Cochrane Reviews show low-quality evidence that task-specific training improves upper limb function. Our feasibility trial showed 56 h of task-specific training over 6 weeks resulted in an increase of a median 6 points on the Action Research Arm test (ARAT), demonstrating the need for more definitive evidence from a larger randomised controlled trial. Task-AT Home is a two-arm, assessor-blinded, multicentre randomised, controlled study, conducted in the home setting.

**Aim:**

The objective is to determine whether task-specific training is a more effective treatment than usual care, for improving upper limb function, amount of upper limb use, and health related quality of life at 6 weeks and 6 months after intervention commencement. Our primary hypothesis is that upper limb function will achieve a ≥ 5 point improvement on the ARAT in the task-specific training group compared to the usual care group, after 6 weeks of intervention.

**Methods:**

Participants living at home, with remaining upper limb deficit, are recruited at 3 months after stroke from sites in NSW and Victoria, Australia. Following baseline assessment, participants are randomised to 6 weeks of either task-specific or usual care intervention, stratified for upper limb function based on the ARAT score. The task-specific group receive 14 h of therapist-led task-specific training plus 42 h of guided self-practice. The primary outcome measure is the ARAT at 6 weeks. Secondary measures include the Motor Activity Log (MAL) at 6 weeks and the ARAT, MAL and EQ5D-5 L at 6 months. Assessments occur at baseline, after 6 weeks of intervention, and at 6 months after intervention commencement. Analysis will be intention to treat using a generalised linear mixed model to report estimated mean differences in scores between the two groups at each timepoint with 95% confidence interval and value of *p*.

**Discussion:**

If the task-specific home-based training programme is more effective than usual care in improving arm function, implementation of the programme into clinical practice would potentially lead to improvements in upper limb function and quality of life for people with stroke.

**Clinical Trial Registration:**

ANZCTR.org.au/ACTRN12617001631392p.aspx

## Introduction

### Background and rationale

Worldwide, 12.2 million people had a stroke in 2019 (1). In Australia, where this study occurs, 1,000 new strokes occur every week and the resultant cost burden is $5 billion a year (2). Problems performing everyday activities of daily living persist 6 months after stroke for 95% of people (3), largely due to not being able to use the arm. Only half of all stroke survivors with an initial affected upper limb regain some useful upper limb function after 6 months (4). Sixty percent of severely impaired and 30% moderately impaired stroke survivors have been reported as having non-functional arms after 6 months (5). Poor recovery of upper limb function is associated with a low health-related quality of life (6, 7).

A Cochrane systematic review of repetitive task training in 2007 (8), most recently updated in 2017 (9), showed a moderate level of evidence for effect of task-specific training on functional ambulation and walking distance, but a low level of evidence for effect on arm and hand function, at ≤6 months after stroke. At the time of conceiving this trial, few studies (10, 11) had directly addressed the hypotheses that task-specific training for the upper limb is more effective than usual care. Several exploratory studies had compared task-specific/functional training to other interventions. For example, Winstein et al. (11) compared 22 participants receiving functional training *in addition to current practice* to 21 receiving *only* usual care, and found an advantage for functional training. Other studies compared task-specific training to alternative treatments such as strength training (12), education (13) or Brunnstrom and Bobath technique (14). About half of the existing studies at the time favoured task-specific training but all were small in sample size (*N* < 53), most had design limitations (e.g., lack of intention to treat analysis, blinded assessor, poor allocation concealment) and several combined the treatment with added interventions so the effect of task-specific training alone could not be estimated. Encouragingly, the EXCITE trial of constraint-induced therapy (CIMT), which contains functional training similar to task-specific training and which was community based, found a positive effect for CIMT on the Wolf Motor Function test at 12 months (primary outcome Wolf Motor Function test) (15).

In a phased approach to developing better treatments, in 2014 we completed a Phase II feasibility RCT with 48 participants (24 task-specific, 23 usual care) using an preliminary version of the design in this protocol (16). Proof-of-concept of task-specific training was demonstrated, with an improvement in median ARAT score (primary outcome measure: range 0–57) of the task-specific group from 8.5 (IQR: 3.0, 24.0) at baseline to 14.5 (3.5, 26.0) at 6 months, compared the usual care group, which had a median score of 4 (3.0, 14.0) which did not change (17). Participants, 85% of whom had moderate–severe impairment, performed a median 157 repetitions per visit plus a median 52 repetitions per day of self-practice, fulfilling the goal of 100–300 reps per day, proving the treatment dose is feasible. 96% of participants rated task-specific training acceptable, 71% rated 1 h of independent practice/day acceptable and 83% reported it improved their arm function. There were no serious adverse events. Based on the results of the feasibility study, we decided to go to the next step to conduct a Phase III trial to investigate effectiveness.

Given that a several existing studies show positive effects of task-specific training compared to other interventions, an important pragmatic question to be answered is whether the new treatment is more beneficial than care routinely delivered now. Consequently, we chose as our comparator group, usual care, where we will not alter treatment intensity or content in the usual care group. Usual care may provide less treatment, but intensity between the two groups will not be matched for the following reasons: (1) matching intensity would not be a true comparison to usual care, because current service provision does not currently offer the proposed intensity of treatment; (2) it is known that increasing intensity of current practice does not deliver the quantum change needed to improve stroke outcome at a population level (18). The importance of our question to health service providers overrides the potential for a difference in intensity of the two treatments. Moreover, if task-specific training is shown to be effective, there will be incentive for increasing intensity when implemented. Treatment intensity will however be carefully recorded.

Since the conception of the present study, in 2016 a randomised controlled trial with 361 participants with stroke, comparing task-oriented rehabilitation to dose-equivalent occupational therapy, or to usual care was published by Winstein et al. (ICARE trial) (19). Findings revealed that a structured, task-oriented rehabilitation program did not significantly improve motor function or recovery beyond either an equivalent or a lower dose of usual care upper extremity rehabilitation. The therapy content was 30 h delivered over 10 weeks and consisted of “intense bouts of task-specific practice, strengthening exercises, shoulder stability/mobility training and motivational enhancements to enable self-confidence and autonomy support to use the stroke-affected arm and hand in valued activities outside the clinic” (19) (Supplementary content, etable2). As the ICARE trial had a similar aim to our study, it is of interest to compare the methods. There are some key differences. We have a more intensive 56 h of intervention over 6 weeks, compared to 30 h over 10 weeks in Winstein 2016 (19). Our study is home-based rather than outpatient based. Primary outcome measure and timepoint also differ. Our primary outcome timepoint (ARAT) is immediately after the intervention at 6 weeks and at 6 months post-randomization. In comparison, in the ICARE trial, the primary outcome (log-transformed Wolf Motor Function Test time score) was measured at 12 months post-randomization. Our study will provide key additional information on the effect of intensity, timing of primary outcome measure and environment. In addition, another small trial was also reported in 2019 (20) comparing task-oriented training to usual care, for the upper limb, with 14 participants allocated to each group. The task-oriented group improved significantly more than usual care on the Wolf Motor Function Test and the Fugl-Meyer upper extremity assessment.

There is a trend to moving inpatient rehabilitation services into the community (21). In Australia in 2020 for example, 42% of services were providing early discharge services compared to 17% in 2016 (22). It follows that much upper limb recovery occurs in the community, after discharge from hospital. At the time of conceiving this study however, a Cochrane review of a range of upper-limb therapies at home (23) showed that we did not yet know if therapy was effective in the home. The likelihood is that home-based treatment will be more beneficial than hospital-based treatment since practice in the person’s own environment is more meaningful (24). In addition, participants might prefer treatment in their home. For example, 96% of participants in our feasibility study (16) found it acceptable. The following two systematic reviews report favourable effects for home rehabilitation in general, though they did not focus particularly on the upper limb. Hillier et al. (25) reported a significant effect in favour of home-based rehabilitation in general compared to centre-based rehabilitation at 6 weeks and 3–6 months post-intervention. Similarly Chi et al. (26) found that home-based rehabilitation led to moderate improvements on physical function in home-dwelling patients with a stroke. To summarise, there are compelling reasons to base therapy at home, but more evidence is still needed.

## Study aims and hypotheses

The primary aim of the Task-AT Home study is to determine if a 6-week task-specific home-based training programme for stroke survivors is more effective than usual care in improving upper limb function and amount of arm use.

The hypotheses relating to this aim are as follows:

1. Upper limb function will achieve a ≥ 5 point improvement on the Action Research Arm Test in the task-specific training group compared to the usual care group, immediately after 6 weeks of intervention (primary outcome).
2. Amount of upper limb use in everyday life will achieve a ≥ 1 point improvement on the Motor Activity Log in the task-specific training group compared to the usual care group immediately after 6 weeks of intervention (secondary outcome).
3. The difference in upper limb function on the Action Research Arm Test between groups will persist to the end of follow up at 6 months after intervention commencement (secondary outcome).

There are 2 secondary aims:

1. Secondary aim 1: To determine if a 6-week task-specific home-based training programme for stroke survivors is more effective than usual care in improving health related quality of life.
  Hypothesis for secondary aim 1: Health related quality of life will achieve a minimally important change of 0.08 point improvement on the EQ5D-5 L in the task-specific training group compared to the usual care group at follow up at 6 months.
2. Secondary aim 2: To identify costs and consequences of both interventions, where consequence is measured by change in health related quality of life.

Supplementary aims:

- Identify potential barriers and possible solutions to implementation of task-specific training. A qualitative study is being conducted using a narrative inquiry methodology. In-depth interviews with eight stroke survivors, three caregivers and four therapists will capture a broad range of perspectives following participation in the training. A detailed description of this study will be reported elsewhere.
- Originally a sub-study was included in our plan to determine changes in motor control in response to training, using kinematics of reach-to-grasp., in the task-specific training group compared to the usual care group. However, due to adjustments that needed to be made to recruitment and funding due to Covid-19, resources were instead redirected to execution of the main trial, so this sub-study could not occur.

## Methods and analysis

### Trial design

This study is a two-arm, assessor-blinded, multicentre randomised controlled trial comparing task-specific treatment to usual care for the arm and hand after stroke. The study will occur in participants’ homes with the interventions delivered by therapists upskilled and employed to deliver the task specific research intervention.

Research personnel responsible for data collection will be blinded to the treatment group to which a participant is assigned. Those involved in the delivery of the treatment will not be blinded. We have also aimed to blind participants as much as possible to group allocation by (a) not informing them deliberately of group allocation – instead they were told they would be visited by a therapist soon and the approximate timeline of this visit. The visit would occur within a week of assessment (if allocated to the task-specific treatment) or in approximately 6 weeks (if allocated to usual care).

The trial is registered with the ANZCTR. Appendix 1 shows key Trial Registration data.

### Study population

Participants with stroke affecting upper limb function will represent the target study population. Participants will be identified during their inpatient stay in stroke wards at hospitals, or *via* community stroke services, in New South Wales (NSW) and Victoria, Australia. In NSW these sites include the Local Health Districts of Hunter New England Health (Kurri Kurri, Maitland, Cessnock and Manning Hospitals, Transitional Aged Care Programme Hunter Valley), Central Coast (Gosford, Long Jetty and Wyong Hospitals, Woy Woy Rehabilitation Centre), Mid North Coast (Port Macquarie, Kempsey and Wauchope Hospitals), Berkley Vale Private Hospital and Mt. Wilga Private Rehabilitation Hospital. In Victoria the sites include health services from Austin Health, Eastern Health and Western Health. Potential participants are identified by therapists working in these organisations and are then screened by the trial manager, at between 2.5 and 3.5 months after stroke. Potential participants who learn about the study *via* social media or the Hunter Medical Research Institute (HMRI) website or study website are also able to self-refer to the study by contacting the research team directly.

Inclusion criteria are diagnosis of primary or recurrent stroke, including stroke caused by focal cerebral infarction (ischemic stroke), intracerebral haemorrhage, subarachnoid haemorrhage and cerebral venous thrombosis (27); discharged home (i.e., permanent address, may include care home/sheltered accommodation); approximately 3 months post stroke (between 2.5 and 3.5 months post stroke); remaining upper limb movement deficit defined as being unable to pick up a 6 mm ball bearing from the tabletop, between index finger and thumb, and place it on a shelf 37 cm above table (item from Action Research Arm Test); informed written consent. Exclusion criteria are upper limb movement deficits attributable to non-stroke pathology; unable to lift hand off lap at all when asked to place hand behind head; severe fixed contractures of elbow or wrist (i.e., grade 4 on the modified Ashworth scale).

### Trial interventions

#### Task-specific intervention

The task-specific intervention will be guided by a detailed protocol (28). The intervention will occur in the participant’s home. In brief, the intervention therapist analyses the whole of the task which is to be trained, e.g., reach-to-grasp., to identify movement components to be prioritised for training and individual movement performance targets to be reached. This will be necessarily different for each participant. The person’s visual attention is directed to regulatory cues in the environment, which are organised to be functionally relevant, individualised and varied, by providing meaningful everyday objects of different sizes, weight and shape, in different positions. The therapist’s role is like a sports coach. He/she uses knowledge of critical biomechanical characteristics of the task to give instructions (by demonstration or verbally) which are concrete and task oriented. Repetitive practice and motor learning principles are used to empower the participant to practice on their own.

Training will be delivered according to an exercise manual containing 142 exercises in words and photographs including variations of the exercises to allow for different levels of difficulty and complexity.

The manual was developed during our previous feasibility trial by a team of therapists during the feasibility trial (Paulette van Vliet, Ailie Turton, Fredreike van Wijck, Paul Cunningham) (28) with consultation with user representatives and local specialist neurophysiotherapists. It describes the underlying principles, clinical objectives and individual exercises to achieve the stated objectives. It also details a menu of the treatment strategies (in words and pictures) and includes variations of the treatment strategies to allow for different levels of difficulty and complexity. A description of the development of the manual is available (28), however the manual itself is under embargo until the completion of the trial.

Participants receive 14 × 1-h visits from a therapist over the 6 weeks (3 visits in weeks 1–3, 2 visits in weeks 4–5, 1 visit during week 6). This will *replace* any usual care training for the upper limb. The intensity of practice within each 1-h session will be dependent on individual participant’s capabilities, but high numbers of repetitions will be encouraged, with the aim of delivering between 100 and 300 repetitions within each 1-h session. Beyond the target of 100–300 reps, participants will do as many repetitions as they can accomplish in 1 h, within limits of fatigue. Any repetition is counted, full range is not required, and repetitions are recorded on a task-specific therapy log form. The number of minutes spent practising each exercise is also recorded on the form, as well as the number of the session (out of 14 sessions), and the total duration of task-specific practice in each session (minutes).

Stretches may be indicated when decreased muscle length is present as a secondary consequence from having had a stroke. When decreased muscle length is interfering with performance of exercises from the task-specific exercise manual, a maximum of 5 min during the 1-h treatment may be spent in stretching and may include short duration stretches of between 8 s to 5 min. Longer duration stretches, of 20–30 min duration, may also be prescribed as part of the patient’s self-managed programme, to be performed outside of their 1 h daily self-practice. Long duration stretches will not be used in the one-to-one treatment sessions with the therapist. The stretches that may be used are described with photographs in a document ‘Upper Limb Stretches’, which is given to the therapists.

##### Self-practice

Participants will be asked to perform in addition to therapy sessions, 1 h/day of self-practice. Adherence is encouraged by joint goal setting, providing a booklet about recovery from stroke emphasising potential for ‘rewiring’ the brain through practice, and using a self-practice log to record repetitions, and the date on which they were performed. The self-practice log is checked by the therapist at the beginning of each therapy session, and the participant is assisted to record the repetitions done if needed. The role of the carer will be to encourage the participant to practice and assist with equipment to enable practice and with recording practice.

##### Usual care intervention

The control group for this trial will receive ‘usual care’. Usual care will be provided according to the usual provision in the Local health District(s)/health services, by the usual staff (not staff employed on the trial). In usual care the frequency and content of therapy is variable according to the individual’s pathway and the range of community services available. Community rehabilitation services may be delivered *via* early supported discharge, day hospital, community-based rehabilitation provided in the home or outpatient rehabilitation (29). Usual care variously consists of facilitation of muscle activity, strengthening exercises, soft tissue and joint mobilisation, positioning, training of sensation, and education (30). While it may include practice of everyday functions, it does not include systematic practice of part-tasks, biomechanical analysis, engaging environmental cues or high numbers of repetitions. Usual care therapists will be requested to indicate any treatment they used for an identified participant on a checklist form listing usual care treatment activities called the Upper Limb Usual Care Therapy Log.

Participants allocated to the usual care group will be provided with a booklet about recovery after stroke, with information about details of the frequency of assessment visits rather than about task-specific training. At the end of a participant’s involvement in the trial, at 6 months after recruitment, the usual care participants will be offered a one-off consultation with a research therapist, in which several exercises from the task-specific manual will be recommended.

### Who delivers treatment, amount of treatment and adherence

Different therapists will deliver the usual care and task-specific interventions. Usual care will be delivered by the usual clinical service physiotherapists and/or occupational therapists. Task-specific intervention will be delivered by a group of therapists employed to deliver the intervention in the trial. Intervention therapists will be trained to deliver task-specific training over a 2-day course including theory, and practice with participants with stroke, prior to delivering the treatment. Further training sessions with participants with stroke, and/or follow-up discussion about intervention, with the trainer are available, if needed. Therapists will also receive instruction in the importance of strictly following the treatment manual.

The treatment duration for the task-specific intervention will be 6 weeks, with 1-h visits occurring 3 times in the first 3 weeks, twice in each of the next 2 weeks, then once in the final week, (tapered to increase self-management).

For each intervention therapist, fidelity to treatment protocol will be assessed. For each therapist, we will aim to assess fidelity on 4 separate occasions. A treatment fidelity checklist will be used. If fidelity to treatment schedule is <90% then further training will be given within the 2 weeks following the fidelity assessment, until the 90% criteria is achieved.

### Outcomes

All outcome measures are performed immediately after the 6 weeks of intervention and at 6 months after intervention commencement by an assessor blind to group allocation. The primary outcome measure is a test of arm function and impairment, the Action Research Arm Test (ARAT) (31), immediately after the 6 weeks of intervention. It consists of 19 items focusing on grasping objects of different shapes and sizes, and gross arm movements. Each item is given an ordinal score of 0, 1, 2, or 3 with higher values indicating better function. The test has high inter-rater and test–retest reliability, good validity and is sensitive to therapy-related changes after stroke. A standardized test protocol will be followed (32).

Secondary outcome measures are (a) the Motor Activity Log (33); (b) the ARAT at 6 months to indicate whether changes occurring at end of 6 weeks are sustained in the longer term; and (c) the EQ5D-5 L (34). The Motor Activity Log is a self-report of quality of upper limb movement and amount of use, to capture the patient perspective. A clinically important mean change on the Motor Activity Log is ≥1; (scale range 0–5). This is a valid, reliable and responsive tool and its scores are strongly correlated with arm accelerometry data (33). The EQ5D-5 L is a standardised measure of health status providing a simple, generic measure of health for clinical and economic appraisal (34). It includes a descriptive system comprising 5 dimensions - mobility, self-care, usual activities, pain/discomfort, and anxiety/depression. Each dimension has 5 levels: no problems, slight problems, moderate problems, severe problems, and extreme problems. It also includes a Visual Analogue scale which records the respondent’s self-rated health on a 20 cm vertical, visual analogue scale with endpoints labelled ‘the best health you can imagine’ and ‘the worst health you can imagine’.

Tertiary outcomes are (a) the Wolf Motor Function test (WMFT) (35); (b) the Fugl-Meyer assessment (upper limb section) (36); (c) the Caregiver Strain Index; and (d) the modified Rankin Scale. The Wolf Motor Function Test assesses a wide range of functional abilities of the upper limb. Fifteen movements both with and without objects are measured according to quality of task performance (graded 0–5) and the time to complete the task. In addition, the number of tasks that can be performed in less than 120 s is assessed. The test is responsive to measuring changes in our target group (15), has high inter-rater reliability, test–retest reliability, internal consistency, and construct validity (35, 37).

The upper limb section of the Fugl-Meyer assessment (FM-UL) (36, 38) is also included as this assessment has been identified by consensus at the Stroke Recovery and Rehabilitation Roundtable (39) as a core measure of sensorimotor recovery that should be included in stroke trials, to allow future pooling of participant data across studies and institutions aiding meta-analyses of completed trials. Caregiver burden will be assessed using the Caregiver Strain Index (40). The CSI is included as we would expect carer burden to decrease with improved upper limb function. The Modified Rankin Scale will be used to measure degree of disability or dependence (41). Finally, to allow determination of costs associated with treatments, the Client Services Receipt Inventory will be used (42).

**Figure 1:**
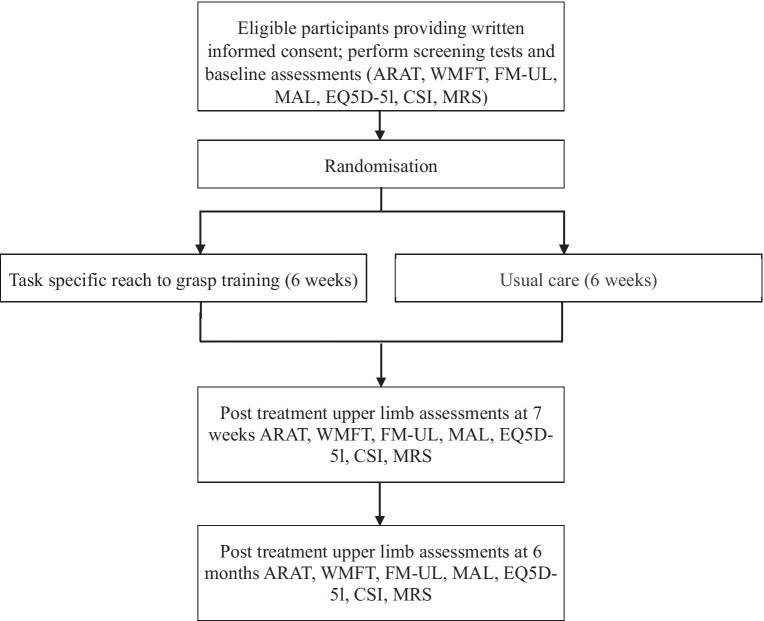
Overview of design and study flow.

The time schedule of enrolment, interventions and assessments, is shown in Figure 1.

### Participant recruitment

Potential participants may be in hospital awaiting discharge or they may already be at home following stroke. In both situations the recruitment procedure will take place over at least two occasions. Screening will take place before consent. First, potential participants will be identified by a clinician, who will check whether potential participants meet the eligibility criteria. If potential participants meet the eligibility criteria, the clinician will make the initial approach to the potential participant, and give the person an invitation letter and Patient Information Sheet (PIS) [approved by the local Research Ethics Committee (REC)]. The clinician will ask the person’s permission to pass their contact details onto a member of the research team. The participant will have at least 24–48 h to read the PIS and to discuss their participation with others outside the research team (e.g., relatives or friends) if they wish. The researcher will go through the PIS and explain the project to make sure the person understands the nature and intensity of the interventions, the randomisation procedure and a participant’s right to withdraw at any time without compromising their care and research governance issues. The researcher will answer questions, confirm the participant’s eligibility and take written informed consent if the participant decides to participate. Those who consent to participate will be assessed at baseline. Following this, participants will be randomised, and the Trial Manager will contact participants by telephone to inform them when their next assessment will be (usual care group) or organise their first therapy appointment and provide the therapist name (task-specific training group). Details of all participants approached for the trial and reason(s) for non-participation (e.g., reason for being ineligible or participant refusal) will be documented.

Strategies being employed for achieving adequate participant enrolment include (a) increasing the number of sites; (b) increased phone and email communication; (c) prompt resolving of local issues relating to recruitment challenges; (d) visiting the sites when possible, and (e) sending a newsletter with news about the trial.

### Study methods

Participants will be stratified according to severity of upper limb impairment, and state (NSW, and Victoria) using randomised permuted blocks (block sizes 4 and 6) in a 1:1 ratio. Baseline severity of upper limb impairment will be categorized using scores on the ARAT (subgroups defined by Morris et al. (43) (Group 1: score 0–3; Group 2 score 4–28; Group 3 score 29–57)). Randomisation will occur after baseline assessment using a computer-generated random allocation sequence, created by the Data Sciences unit at the HMRI. The Trial Manager will randomise each participant using REDCap, then inform the therapist of the treatment allocation and book assessment appointments.

Outcome assessors will be blinded to the treatment group to which a participant is assigned. Owing to the nature of this behavioural intervention, therapists delivering treatment cannot be blinded. Participants will be instructed in their treatment but will be blinded to the treatment group.

### Data collection

Data will be collected and stored using a web-based database REDCap, hosted by HMRI on a secure server. After giving consent, a baseline assessment will be conducted for the participant. As well as the outcome measures, demographic data will be collected at baseline including:

- Whether thrombolysis or clot retrieval was used (if this information is available)
- National Institute of Health Stroke Scale (NIHSS)
- Assessment of upper limb sensory deficit including proprioception (Wrist Position Sense Test (44)) and touch (Tactile Discrimination Test (45))
- Ability to perform voluntary finger extension (within the Fugl-Meyer Assessment, by test item 25 -‘extend all fingers’)
- The Montreal Cognitive Assessment (MoCA) (46), a rapid screening instrument for mild cognitive dysfunction. It assesses different cognitive domains: attention and concentration, executive functions, memory, language, visuoconstructional skills, conceptual thinking, calculations, and orientation
- Self-efficacy using the Stroke Self-Efficacy Questionnaire (47)
- Depression using the Hospital Anxiety and Depression Scale (HADS) (48) or the Stroke Aphasic Depression Questionnaire (SADQ) (49). To determine which of these scales is used, language is assessed using the Sheffield Screening test for Language Disorders (receptive language subsection) (50). If the participant scores less than 9 out of 9 on the receptive subsection, the SADQ is administered, instead of the HADS
- Presence of vascular risk factors, comorbid conditions such as cognitive decline, arthritis and renal disease, other neurological disease, and type of stroke -ischaemic/haemorrhagic, lacunar/large artery/other, cortical/subcortical, whether thrombolytic drugs were used, side of hemiplegia, handedness whether able to walk independently at stroke onset, and whether active hand movement was present at stroke onset.

Assessments will be conducted at home or in a designated clinic room in a hospital or in a research institute attached to a hospital by blinded assessors who are different to the therapists delivering treatment. Outcome assessors receive 1.5 days training with CI Paulette van Vliet and/or CI Leanne Carey and the local trial manager, including tutorials and practice of each assessment, and on the wider context of the trial, adhering to trial methods (e.g., avoiding being unblinded), and completing the participant case report form. This is followed by two actual participant assessments supervised by an experienced assessor.

Every effort is made to complete the follow-up assessments at 6 weeks and 6 months. In the event that the participant is unable to complete all the assessments, priority is given to at least attempting to obtain the primary outcome measure the ARAT, and then the MAL and the EQ5D-5 L, if possible.

### Modifications due to Covid-19

In March 2020, in NSW, recruitment, assessments and therapy were temporarily stopped for 4 months whilst modified procedures were developed and piloted to adjust to the presence of Covid-19 in the community. Modifications to the assessment and therapy procedures (listed below) were adopted and then recruitment was restarted at sites that were not under Covid-19 restrictions. In Victoria, restrictions were experienced from March 2020 to March 2022. In addition, state and site-specific adjustments were made. For example, in accordance with advice from NSW Health, the following will also apply to all research staff in contact with participants in this study: COVID-19 Vaccination will be mandatory for all NSW staff with first vaccination required by 30 September 2021 and second vaccination by 30 November 2021 in order to continue to work for NSW Health. Evidence of vaccination for the research staff in contact with participants will be provided. Modifications were approved by the ethics committee.

A further pause of recruitment, assessments and therapy occurred in NSW in September 2021 due to NSW Government and University of Newcastle restrictions regarding contact with participants and conducting field work. Due to the adverse effect of Covid-19 on recruitment, as well as lower than expected recruitment rates prior to the advent of Covid-19, during the data monitoring committees of 2020 and 2021, a more realistic expected number that could be recruited was proposed, which was 100 participants.

#### Changes to therapy procedures

Changes have been made to the procedures for conducting the task-specific therapy sessions, in order to adapt to the Covid-19 pandemic, as follows:

1. COVID19 screening questions are applied prior to the visit. If the person indicates that in the last 14 days they have been overseas, in contact with someone who has returned from overseas, in contact with any person confirmed as Covid-19 positive, in contact with someone with suspected case of Covid-19, or suffering respiratory symptoms, therapy visits are postponed until the participant’s symptoms abate and they test negative for COVID
2. If there is a pause in therapy due to any of the reasons in point 1 above, the missed treatment sessions can be added after the 6-week treatment period, up to a period of 8 weeks from the first treatment session
3. Therapists are required to have completed minimum level education and training in Infection Prevention and Control related to COVID-19 and provide certificate; and are requested to a have a recent influenza vaccination, and provide evidence to the trial manager if they have
4. All contents of the therapy kits (including the suitcases) which cannot be cleaned effectively have been replaced with versions of the item that can be thoroughly cleaned
5. Physically distance as much as possible during the visit so that at least 1.5 M is between therapist and carer/participant
6. Disposing of waste in the person’s home before leaving using the usual waste streams
7. Therapists have been provided with gloves, masks, disinfectant cleaning spray and alcohol/detergent wipes, and a thermometer for temperature screening. In case of a period of high community transmission of COVID19 occurring, additional PPE will be provided, including disposable gowns and eye protection
8. Training sessions maybe conducted *via* video call, *via* zoom, if a participant does report as having Covid-like or respiratory symptoms, or is confirmed as having COVID-19, but feels they can still do upper limb exercises, until such time as they can be seen in person again.

#### Changes to assessment procedures

Changes have been made to the procedures for conducting the baseline measures and outcome assessments, in order to adapt to the Covid-19 pandemic, as follows:

1. COVID19 screening questions are applied prior to the visit. If the person indicates that in the last 14 days they have been overseas, in contact with someone who has returned from overseas, in contact with person confirmed as Covid-19 positive, in contact with someone with suspected case of Covid-19, or suffering respiratory symptoms, assessment visits are postponed until the participant’s symptoms abate and they test negative for COVID
2. Assessors are required to have completed minimum level education and training in Infection Prevention and Control related to COVID-19 and provide certificate; and are requested to a have a recent influenza vaccination, and provide evidence if they have
3. All contents of the assessment kits (including the suitcases) which cannot be cleaned effectively have been replaced with versions of the item that can be thoroughly cleaned
4. Physically distance as much as possible during the visit so that at least 1.5 M is between assessor and carer/participant
5. Disposing of waste in the person’s home before leaving using the usual waste streams
6. Assessors have been provided with gloves, masks, disinfectant cleaning spray and alcohol/detergent wipes, and a thermometer for temperature screening. In case of a period of high community transmission of COVID19 occurring, additional PPE has been provided, including disposable gowns and eye protection
7. During periods of high community transmission of COVID19, in order to limit exposure of participants, and assessors to infectious agents, assessment items that can be conducted by phone, are being conducted by phone. The assessments being conducted by phone are:

- Modified Rankin Scale
- Motor Activity Log
- National Institute of Stroke Scale. Items 9 and 10
- Client Services receipt Inventory

8. During periods of high community transmission of COVID19, the baseline assessment has been split into 3 sections which are performed at different days/times (within 1 week of each other), in order to limit exposure of participants, researchers or staff to infectious agents.

### Data management

The end of the trial for an individual participant is defined as completion of the 6-month follow-up. The definition of the end of the trial as a whole is the date when all participants have completed their final assessment at follow-up or have been lost to follow-up.

The Trial Manager/research assistant at each site will enter data into REDCap. The Data Sciences unit at HMRI will develop and upload the randomisation schema; develop and maintain the data management system; develop a data management plan for the data; prepare and clean the data for analysis; write the statistical analysis plan and conduct the statistical analysis.

The Trial Manager/research assistant will collect information about the number of eligible participants approached, recruit the participants, including obtaining consent from those who choose to join the study and will ‘track’ each participant to ensure data collection at the designated times.

Study staff will review of data for accuracy during data entry, with data quality aided by logging of data entry and changes (with comments). The Trial Manager/research assistant will also be responsible for the secure storage of data coming from participants across research sites. Paper records of assessment scores and questionnaires will be anonymised using code identifier and kept in a locked filing cabinet in an office that is locked when not occupied by the research team.

### Sample size

We aim to detect a minimal clinically important difference of 5 points on the ARAT, which derives from findings of van der Lee et al. (51), who reported a difference of 5.7 for a mildly impaired population. The clinically important mean change for the ARAT for different severity groups are not yet published. We have adjusted to 5 to reflect the inclusion of moderate to severe impairment in our expected population, [our previous feasibility study population included 37% participants with severe (i.e., ARAT 0–3) impairment] (17). Based on observed values from our feasibility study, i.e., a pooled standard deviation on the ARAT of 18 and a correlation between baseline and follow-up of 0.72, 300 participants (150 per group) will provide the study with 80% power to detect the 5 point change on the ARAT at the 5% significance level (two-tailed test), allowing for a 20% loss to follow-up. Using similar assumptions, the study would have 80% power to detect differences between groups of 0.3 SDs on the WMFT, MAL, and the Caregiver Strain Index.

### Statistical analysis

Intention-to-treat (ITT) and per protocol (PP) analyses will be conducted. The ITT population will include all subjects who are randomised whereas the PP population will include those subjects who received 90% allocated treatment. The primary outcome measure (ARAT at the end of intervention at 6 weeks) will be analysed using analysis of covariance fitted within a Generalised Linear Mixed Model (GLMM) framework to adjust for the repeated measurements on individuals. The outcome in the model will be the individual’s ARAT (immediately after 6 weeks of intervention/6 months after intervention commencement); fixed effects in the model will be group, time and the interaction of time by group, baseline ARAT score and stratification variables (site and severity of upper limb impairment). A random effect for subject will be included. Secondary outcome measures of MAL, WMFT, FMA, EQ5D-5 L, CSI, and mRS will be analysed using the same approach as used to test the primary outcome, with appropriate distributional families depending on the type of outcome. Pre-specified sub-group analysis: We will conduct sub-group analyses to determine differential effects of the intervention in participants with voluntary finger extension (no extension, partial, or full extension) at 3 months post stroke. The GLMM regression model described above will be fitted within each of the sub-groups. No formal tests of significant interactions will be assessed for these models, since the study is not powered for these tests. The primary analysis will take place when follow-up is complete for all recruited participants. No formal interim analysis is planned.

### Data monitoring

An independent Data Monitoring and Safety Committee (DMC) has been formed, consisting of an expert statistician uninvolved with the trial, and a researcher in the field who is also an experienced physiotherapist, uninvolved with the trial. The role of the DMC is to monitor the data from the trial and to make recommendations to the Chief Investigator and trial manager and to consider any safety issues for the trial. The DMC is independent from the sponsor and competing interests. Meetings will be held during recruitment, and then when recruitment reaches *n* ≥ 50. The trial team will prepare the following reports for that meeting:

- Recruitment report
- Demographics of participants
- Number of participants completed, number of participants to follow-up, number of participants lost to follow-up
- Number of participant ARAT (primary outcome) assessments performed at baseline, 6 weeks and 6 months
- Adverse events
- Adherence data.

Following the meetings, a report will be provided to the Chief Investigator, by the chair of the DMC, and the Chief Investigator will report the findings to the Trial Management Committee. When the participant number exceeds 50, a report will be provided for the next DMC meeting of the outcome data, unblinded, by the trial statistician. At meetings where the unblinded outcome data is discussed, there will be both an open and a closed meeting, with the closed meeting including the DMC, the trial manager, and trial statistician if required (and not the Chief Investigator).

### Expected adverse events and safety reporting

There are adverse events that are expected in the stroke population that are not related to the task-specific intervention. These are: death, further stroke, cardiovascular conditions and illnesses relating to old age, and development of secondary complications: such as joint contractures. Poor arm function after stroke is also associated with poor balance and falls. While there is low risk of shoulder pain or hand pain related to the task specific intervention and injury to the hand is not expected as a result of the intervention, musculoskeletal adverse events, in addition to common serious adverse events will be monitored as part of the trial.

In the event of shoulder or hand pain occurring in participants in either group of the trial the clinical physiotherapists or occupational therapists would, as part of their role, expect to treat such symptoms or refer the trial participant to another service. Incidence of expected and unexpected adverse events will be collected by the research physiotherapist/occupational therapist and reported to the Data Monitoring Committee.

### Ethics and dissemination

Ethics review of the protocol for the trial and other trial related essential documents (e.g., Participant Information Statement and consent form) will be carried out by the main Human Research Ethics Committee (HREC), Hunter New England REC, the University of Newcastle Human Ethics Committee, and also site RECs, including Central Coast Local Health District REC, Mid North Coast Local Health District REC, Austin Health REC, La Trobe University HREC, Eastern Health HREC, Western Health HREC.

Any amendments to these documents, will be submitted to the REC for approval prior to implementation. Important protocol modifications will be communicated to the RECs, investigators, sponsor, and Trial Management Committee.

The process for informed consent is as follows. Potential participants will be identified by a clinician, who will check whether potential participants meet the eligibility criteria. If potential participants meet the eligibility criteria, the clinician will make the initial approach to the potential participant and give the person an invitation letter and Patient Information Sheet (PIS; approved by the REC, see Appendix 2). The clinician will ask the person’s permission to pass their contact details onto a member of the research team. The participant will have at least 24–48 h to read the PIS and to discuss their participation with others outside the research team (e.g., relatives or friends) if they wish. The researcher will go through the PIS and explain the project to make sure the person understands the nature and intensity of the interventions, the randomisation procedure and a participant’s right to withdraw at any time without compromising their care and research governance issues. The researcher will answer questions, confirm the participant’s eligibility, and take written informed consent if the participant decides to participate. A model consent form is included at Appendix 3.

### Data protection and participant confidentiality

Data will be collected and retained in accordance with the Australian Code for the Responsible Conduct of Research guidelines (52). Data will be entered onto a purpose designed database and data validation and cleaning will be carried out throughout the trial. Trial data will be entered directly into the study database *via* secure restricted internet access.

All study documentation will be retained in a secure location during the conduct of the study and for 15 years after the end of the study, when all participant identifiable paper records will be destroyed by confidential means. Where trial related information is documented in the medical records, these records will be identified by a label bearing the name and duration of the trial. Australian Good Clinical Practice research guidelines, relevant ‘meta’-data about the trial and the full dataset, but without any participant identifiers other than the unique participant identifier, will be held for up to 15 years on a secure University server. A secure electronic ‘key’ with a unique participant identifier, and key personal identifiers (e.g., name, date of birth and medical record number) will also be held indefinitely, but in a separate file and in a physically different location. These will be retained because of the potential for the raw data to be used subsequently for secondary research.

Data will not be made available for sharing until after publication of the main results of the study. Thereafter, anonymised individual participant data will be made available for secondary research, conditional on assurance from the secondary researcher that the proposed use of the data is compliant with the Australian Good Clinical Practice research guidelines regarding scientific quality, ethical requirements and value for money. A minimum requirement with respect to scientific quality will be a publicly available pre-specified protocol describing the purpose, methods and analysis of the secondary research, e.g., a protocol for a Cochrane systematic review. The second file containing participant identifiers would only be made available for record linkage or a similar purpose, subject to confirmation that the secondary research protocol has been approved by a HREC or other similar, approved ethics review body.

The findings will be disseminated by usual academic channels, i.e., presentation at international meetings, as well as by peer-reviewed publications and through participant organisations and newsletters to participants, where available. No commercially exploitable findings are anticipated.

#### Sponsor contact information

The trial Sponsor is the University of Newcastle, University Drive, Callaghan, NSW, 2308 Australia, and the sponsor’s reference for the study is H-2018-0256.

#### Funder

The trial was funded by project grant number APP1129008 from the National Health and Medical Research Council. This funding source had no role in the design of this study and will not have any role during its execution, analyses, interpretation of the data, or decision to submit results.

### Limitations to the study

In this study protocol, it is acknowledged that in making certain considered choices regarding our research plan, these choices, however well-reasoned, may give rise to other potential limitations, so we would like to note these here. Firstly, we chose to use a broad-based inclusion criteria, in order to make our results applicable to the wider stroke population. So we decided not to exclude people with no finger extension at time of recruitment, neglect/inattention and dyskinesia. The presence of these factors may impede the effects of rehabilitation, but since we were not aware of evidence that task-specific training might not still work with these people, we allowed them to be included. Secondly, for those participants with low baseline scores on the ARAT or no finger extension at the time of recruitment, which would be predictive of poor upper limb recovery, it could be argued that these participants could be closely monitored with more frequent assessments, in order to ascertain whether continuation of therapy is justified. To deal with potential limited recovery of finger extension, in this study we have included two other assessments as well as the ARAT, namely the WMFT and the FMA, which allow improvements to be measured in other movements involving the shoulder, elbow and wrist, even if finger extension has not recovered. However, in planning future studies, it would be good to include a mechanism to justify continuation of therapy. Thirdly, due to finite resources, which were under further pressure due to the effects of Covid, it was not possible to periodically check the fidelity of each individual assessor’s assessment technique, after their initial competence was checked after training. Assessors did receive a thorough training for their role, including supervised assessments with participants. Additional tutorials on the FMA and WMFT were held by zoom as needed, video materials of these assessments being done with patients and scores were provided for extra practice/confirmation and the trainers were made available to assessors for further input when requested. Also, when assessments were returned to the trial manager, they were thoroughly checked and any anomalies followed up. However we acknowledge that periodic checking of assessor technique is desirable in future trials.

### Protocol amendments

The protocol was updated twice following trial registration. Both updates occurred after enrolment was initiated on 12/12/2018 as outlined below.

Post-enrolment revision #1 (randomized accrual at this time = 12): The first revision was made 27/08/20.

New recruitment sites Berkley Vale Private Hospital and Mt. Wilga Private Rehabilitation Hospital were added. Outcome measures were added: the Stroke Self-Efficacy Questionnaire, Sheffield Screening Test Receptive Skills, Stroke Aphasic Depression Questionnaire and Hospital Anxiety and Depression Scale. A stretching document was produced to guide the use of stretches during the task specific visits. An ‘Upper Limb Usual Care Therapy Log’ form was developed for treatment of participants allocated to receive usual care. A column for ‘time’ was added to the Therapist’s ‘Task Specific Therapy Log’ form for each task specific exercise. Extra clarification of surrounding time between baseline and task specific therapy, with task specific therapy starting 1 week after baseline, and that participant assessments should occur within 3 days either side of an appointment which is cancelled. A document describing the assessment of fidelity to treatment intervention was developed. Adjustments to assessment and therapy procedures for Covid19 were added.

Post-enrolment revision #2 (randomized accrual at this time = 31). The second revision was made 15/09/21.

New recruitment sites, the Central Coast Local Health District and the Mid North Coast Local Health District of NSW, and Eastern Health and Western Health, in Victoria. Further advice re COVID-19 Vaccination will be mandatory for all NSW staff with first vaccination was added.

## Ethics statement

The studies involving human participants were reviewed and approved by Hunter New England Health Research Ethics Committee. The patients/participants provided their written informed consent to participate in this study.

## Author contributions

PV, AT, and LC conceived of the study. PV, AT, LC, GK, MT, AS, SM, BD-B, and SO formulated the study design. PV, AT, LC, GK, MT, AS, and SM are grant holders. CO and KP provide statistical expertise and will conduct the statistical analysis. All authors contributed to the article and approved the submitted version.

## Conflict of interest

The authors declare that the research was conducted in the absence of any commercial or financial relationships that could be construed as a potential conflict of interest.

## Publisher’s note

All claims expressed in this article are solely those of the authors and do not necessarily represent those of their affiliated organizations, or those of the publisher, the editors and the reviewers. Any product that may be evaluated in this article, or claim that may be made by its manufacturer, is not guaranteed or endorsed by the publisher.

## Supplementary material

APPENDIX 1 Trial registration data.

APPENDIX 2 Participant Information Sheet.

APPENDIX 3 Participant Consent Form.

## References

[1] World Stroke Organization. World Stroke Organisation Annual Report. Geneva: World Stroke Organization (2021).

[2] Australian Institute of Health and Welfare. Australian Burden of Disease Study: Impact and Causes of Illness and Death in Australia. Australian Government. (2019).

[3] Kong KH Chua K Lee J. Recovery of upper limb dexterity in patients more than 1 year after stroke: frequency, clinical correlates and predictors. NeuroRehabilitation. (2011) 28:105–11. doi: 10.3233/NRE-2011-0639, PMID: 21447911

[4] Kwakkel G Kollen BJ Vandergrond J AJH P. Probability of regaining dexterity in the flaccid upper limb. Stroke. (2003) 34:2181–6. doi: 10.1161/01.STR.0000087172.16305.CD, PMID: 12907818

[5] Houwink A Nijland RH Guerts AC Kwakkel G. Functional recovery of the paretic upper limb after stroke: who regains hand capacity? Arch Phys Med Rehabil. (2013) 94:839–44. doi: 10.1016/j.apmr.2012.11.031, PMID: 23201317

[6] Franceschini M Porta FL Agosti M Massucci M. Is health-related quality of life of stoke patients influenced by neurological impairments at one year after stroke? Eur J Phys Rehabil Med. (2010) 46:389–99. PMID: 20927005

[7] Nichols-Larsen DS Clark PC Zeringue A. Factors influencing stroke survivor's quality of life during subacute recovery. Stroke. (2005) 36:1480–4. doi: 10.1161/01.STR.0000170706.13595.4f 15947263

[8] French B Thomas LH Leathley MJ Sutton CJ McAdam J Forster A . Repetitive task training for improving functional ability after stroke. Cochrane Database Syst Rev. (2007) 11:CD006073. doi: 10.1002/14651858.CD006073.pub2 17943883

[9] Thomas LH French B Coupe J McMahon N Connell L Harrison J . Repetitive task training for improving functional ability after stroke. A major update of a Cochrane review. Stroke. (2017) 48:e102–3. doi: 10.1161/STROKEAHA.117.016503 PMC6464929 27841442

[10] Turton AJ Fraser CM. The use of home therapy programmes for improving recovery of the upper limb following stroke. Br J Occup Ther. (1990) 53:457–62. doi: 10.1177/030802269005301104

[11] Winstein CJ Rose DK Tan SM Lewthwaite R Chui HC Azen SP. A randomized controlled comparison of upper-extremity rehabilitation strategies in acute stroke: a pilot study of immediate and long-term outcomes. Arch Phys Med Rehabil. (2004) 85:620–8. doi: 10.1016/j.apmr.2003.06.027, PMID: 15083439

[12] Thielmann GT Dean CM Gentile AM. Rehabilitation of reaching after stroke: task-related training versus progressive resisted exercise. Arch Phys Med Rehabil. (2004) 85:1613–8. doi: 10.1016/j.apmr.2004.01.028 15468020

[13] Harris JE Eng JJ Miller WC Dawson AS. A self-administered graded repetitive arm supplementary program (GRASP) improves arm fucntion during inpaitent stroke rehabilitation: a multi-site randomzed controlled trial. Stroke. (2009) 40:2123–8. doi: 10.1161/STROKEAHA.108.544585, PMID: 19359633

[14] Arya KN Verma R Garg RK Sharma VP Argarwal M Arggarwal G. Meaningful task-specific training (MTST) for stroke rehabilitation: a randomized controlled trial. Top Stroke Rehabil. (2012) 19:193–211. doi: 10.1310/tsr1903-193, PMID: 22668675

[15] Wolf SL Winstein CJ Miller JP Taub E Uswatte G Morris D . Effect of constraint-induced movement therapy on upper extremity function 3 to 9 months after stroke. J Am Med Assoc. (2006) 296:2095–104. doi: 10.1001/jama.296.17.2095, PMID: 17077374

[16] Turton A Cuningham P Heron E Fv W Sackley C Rogers C . A feasibility study for a randomised controlled trial of home based reach to grasp training for people after stroke. Trials. (2013) 14:109. doi: 10.1186/1745-6215-14-109, PMID: 23782653 PMC3675391

[17] Turton A Cunningham P Fv W Smart H Rogers CA Sackley CM . Home-based reach-to-grasp training for people after stroke is feasible: a pilot randomised controlled trial. Clin Rehabil. (2017) 31:891–903. doi: 10.1177/0269215516661751, PMID: 27470470

[18] French B Leathley M Sutton C McAdam J Thomas L Forster A . A systematic review of repetitive functional task practice with modelling of resource use, costs and effectiveness. Health Technol Assess. (2008) 12:1–117. doi: 10.3310/hta12300 18547501

[19] Winstein CJ Wolf SL Dromerick AW Lane CJ Nelsen MA Lewthwaite R . Effect of a task-oriented rehabilitation program on upper extremity recovery following motor stroke: the ICARE randomized clinical trial. J Am Med Assoc. (2016) 315:571–81. doi: 10.1001/jama.2016.0276, PMID: 26864411 PMC4795962

[20] Thant AA Wanpen S Nualnetr N Puntumeta R Chatchawan U Hla KM . Effects of task-oriented training on upper extremity functional performance in patients with sub-acute stroke: a randomized controlled trial. J Phys Ther Sci. (2019) 31:82–7. doi: 10.1589/jpts.31.82, PMID: 30774211 PMC6348189

[21] Langhorne P Baylan S Early supported discharge Trialists. Early supported discharge services for people with acute stroke. Cochrane Database Syst Rev. (2017) 7:CD000443. doi: 10.1002/14651858.CD000443.pub4 28703869 PMC6483472

[22] National Stroke Foundation Audit Rehabilitation Services Report (2020).

[23] Coupar F vanVliet P Legg L Pollock A Sackley C. Home-based therapy programmes for upper limb functional recovery following stroke. Cochrane Database Syst Rev. (2012) 2012:CD006755. doi: 10.1002/14651858.CD006755.pub2 22592715 PMC6464926

[24] Proteau L Marteniuk RG Levesque L. A sensorimotor basis for motor learning: evidence indicating specificity of practice. Q J Exp Psychol. (1992) 44:557–75. doi: 10.1080/14640749208401298, PMID: 1631322

[25] Hillier S Inglis-Jassiem G. Rehabilitation for community-dwelling people with stroke: home or Centre based? A systematic review. Int J Stroke. (2010) 5:178–86. doi: 10.1111/j.1747-4949.2010.00427.x 20536615

[26] Chi NF Huang Y-C Chiu H-Y Chang H-J Huang H-C. Systematic review and meta-analysis of home-based rehabilitation on improving physical function among home-dwelling patients with a stroke. Arch Phys Med Rehabil. (2020) 101:359–73. doi: 10.1016/j.apmr.2019.10.181, PMID: 31689417

[27] Sacco RL Kasner SE Broderick JP Connors JJB Culebras A Elkind MSV . An updated definition of stroke for the 21st century: a statement for healthcare professionals from the American Heart Association/American Stroke Association. Stroke. (2013) 44:2064–89. doi: 10.1161/STR.0b013e318296aeca, PMID: 23652265 PMC11078537

[28] Cunningham P Turton A Wijke FV Vliet PV. Task-specific reach-to-grasp training after stroke: development and description of the home-based intervention. Clin Rehabil. (2016) 30:731–40. doi: 10.1177/0269215515603438, PMID: 26337625

[29] National Stroke Foundation National Stroke Services Rehabilitation Report (2012).

[30] Donaldson C Tallis RC Pomeroy VM. A treatment schedule of conventional physical therapy provided to enhance upper limb sensorimotor recovery after stroke: expert criterion validity and intra-rater reliability. Physiotherapy. (2009) 95:110–9. doi: 10.1016/j.physio.2008.11.005, PMID: 19627692

[31] Hseih C Hsueh IP Chiang F-M Lin P-H. Inter-rater reliability of the action research arm test in stroke patients. Age Ageing. (1998) 27:107–13. doi: 10.1093/ageing/27.2.107, PMID: 16296669

[32] Yozbatiran N Der-Yeghiaian L Cramer SC. A standardized approach to performing the action research arm test. Neurorehabil Neural Repair. (2008) 22:78–90. doi: 10.1177/1545968307305353 17704352

[33] Uswatte G Taub E Morris D Vignolo M McCulloch K. Reliability and validity of the upper-extremity motor activity Log-14 for measuring real-world arm use. Stroke. (2005) 36:2493–6. doi: 10.1161/01.STR.0000185928.90848.2e, PMID: 16224078

[34] Kim SH Kim HJ Si L Jo M-W. Comparing the psychometric properties of the EQ-5D-3L and EQ-5D-5L in cancer patients in Korea. Qual Life Res. (2012) 21:1065–73. doi: 10.1007/s11136-011-0018-1, PMID: 21947656

[35] Wolf S Catlin PA Ellis M Archer AL Morgan B Piacentino A. Assessing Wolf Motor function test as outcome measure for research in patients after stroke. Stroke. (2001) 32:1635–9. doi: 10.1161/01.STR.32.7.1635 11441212

[36] Fugl-Meyer AR Jaasko L Leyman I Olsson S Steglind S. The post-stroke hemiplegic patient: a method for evaluation of physical performance. Scand J Rehabil Med. (1975) 7:13–31. PMID: 1135616

[37] Morris DM Uswatte G Crago JE Cook EW Taub E. The reliability of the Wolf Motor function test for assessing upper extremity function after stroke. Arch Phys Med Rehabil. (2001) 82:750–5. doi: 10.1053/apmr.2001.23183, PMID: 11387578

[38] See J Dodakian L Chou C Chan V McKenzie A Reinkensmeyer DJ . A standardized approach to the Fugl-Meyer assessment and its implications for clinical trials. Neurorehabil Neural Repair. (2013) 27:732–41. doi: 10.1177/1545968313491000, PMID: 23774125

[39] Kwakkel G Lannin NA Borschmann K English C Ali M Churilov L . Standardized measurement of sensorimotor recovery in stroke trials: consensus-based core recommendations from the stroke recovery and rehabilitation roundtable. Int J Stroke. (2017) 12:451–61. doi: 10.1177/1747493017711813, PMID: 28697709

[40] Robinson BC. Validation of a caregiver strain index. J Gerontol. (1983) 38:344–8. doi: 10.1093/geronj/38.3.344 6841931

[41] Banks JL Marotta CA. Outcomes validity and reliability of the modified Rankin scale: implications for stroke clinical trials. Stroke. (2007) 38:1091–6. doi: 10.1161/01.STR.0000258355.23810.c6, PMID: 17272767

[42] Beecham J Knapp M. Costing psychiatric interventions In: Thornicraft G, editor. Measuring Mental Health Needs. London: Royal College of Psychiatrists. (2001). 200–24.

[43] Morris J Fv W Joice S Ogston S Cole I MacWalter R. A comparison of bilateral and unilateral upper limb task training in early post stroke rehabilitation: a randomised controlled trial. Arch Phys Med Rehabil. (2008) 89:1237–45. doi: 10.1016/j.apmr.2007.11.039, PMID: 18586126

[44] Carey L Oke LE Matyas TA. Impaired limb position sense after stroke: a quantitative test for clinical use. Arch Phys Med Rehabil. (1996) 77:1271–8. doi: 10.1016/S0003-9993(96)90192-6, PMID: 8976311

[45] Carey LM Oke LE Matyas TA. Impaired touch discrimination after stroke: a quantitative test neurorehabilitation and neural repair (1997) 11:219–32. doi: 10.1177/154596839701100404,

[46] Nasreddine Z Phillips NA Bédirian V Charbonneau S Whitehead V Collin I . The Montreal cognitive assessment, MoCA: a brief screening tool for mild cognitive impairment. J Am Geriatr Soc. (2005) 53:695–9. doi: 10.1111/j.1532-5415.2005.53221.x, PMID: 15817019

[47] Jones F Partridge C Reid F. The stroke self-efficacy questionnaire: measuring individual confidence in functional performance after stroke. J Clin Nurs. (2008) 17:244–52. doi: 10.1111/j.1365-2702.2008.02333.x, PMID: 18578800

[48] Zigmond AS Snaith RP. The hospital anxiety and depression scale. Acta Psychiatr Scand. (1983) 67:361–70. doi: 10.1111/j.1600-0447.1983.tb09716.x 6880820

[49] Sutcliffe LM Lincoln NB. The assessment of depression in aphasic stroke patients: the development of the stroke aphasic depression questionnaire. Clin Rehabil. (1998) 12:506–13. doi: 10.1191/026921598672167702 9869254

[50] Syder D Body R Parker M Body M. Sheffield Screening Test for Acquired Language Disorders. Windsor: NFER Nelson (1993).

[51] van der Lee J Beckerman H Lankhorst GJ Bouter LM. The responsiveness of the action research arm test and the Fugl-Meyer assessment scale in chronic stroke patients journal of rehabilitation medicine (2001) 33:110–3. doi: 10.1080/165019701750165916, 11482350

[52] National Health and Medical Research Council. Australian Code for the Responsible Conduct of Research. (2018).

