# Supplementary material for: Task-specific training versus usual care to improve upper limb function after stroke: the “Task-AT Home” randomised controlled trial protocol
Source: Front Neurol. 2023 Jun 29;14:1140017. doi: 10.3389/fneur.2023.1140017 (PMC10345498; doi:10.3389/fneur.2023.1140017)
Supplement: Supplementary file 1 [file Data_Sheet_1.pdf]

Appendices:

Appendix 1 – Trial registration data

Appendix 2 – Participant Information Sheet

Appendix 3 – Participant Consent Form

Trial registration data

Primary registry and trial identifying number	ANZCTR.org.au/ACTRN12617001631392p.aspx
Date of registration in primary registry	15/12/2017
Source(s) of monetary or material support	National Health and Medical Research Council Project grant<br><br>APP1129008; Funding period November 2016- February 2023
Primary sponsor	University of Newcastle, Australia
Contact for public queries	Paulette.vanvliet@newcastle.edu.au
Contact for scientific queries	Paulette.vanvliet@newcastle.edu.au
Public title	Task-AT Home
Scientific title	Improving arm function after stroke using task-specific training
Countries of recruitment	Australia
Health condition(s) or problem(s) studied	Upper limb recovery following stroke
Intervention(s)	Task-specific training
Active comparator:	Usual care
Key inclusion and exclusion criteria	**Inclusion criteria:**<br>A participant may enter the study if ALL of the following apply<br><br>1. Diagnosis of primary or recurrent stroke<br>2. Participant discharged home (i.e. permanent address, may include care home/sheltered accommodation)<br>3. Participant at approximately 3 months post stroke (between 2.5 and 3.5 months post stroke)

	4. Has remaining upper limb movement deficit defined as being unable to pick up a 6mm ball bearing from the table top, between index finger and thumb, and place it on a shelf 37 cm above table (item from Action Research Arm Test)<br>5. Informed written consent<br>**Exclusion criteria:**<br>A participant will not enter the study if ANY of the following apply<br>1. Upper limb movement deficits attributable to non-stroke pathology<br>2. Unable to lift hand off lap at all when asked to place hand behind head<br>3. Severe fixed contractures of elbow or wrist (i.e. grade 4 on the modified Ashworth scale)
Study type	Randomised controlled trial
Allocation	two-arm, assessor-blinded, multicentre randomised
Primary purpose	Compare interventions
Phase	III
Date of first enrolment	12/12/2018
Target sample size	300 (revised to 100 due to Covid)
Recruitment status	Recruiting
Primary outcome(s)	Action Research Arm Test
Key secondary outcomes	Motor Activity Log, EQ5D-5l, Wolf Motor Function Test, Fugl-Meyer Assessment, Care giver strain index, Modified Rankin Scale

Research and Innovation Division
The University of Newcastle
Callaghan NSW 2308

Participant Information Sheet

Full Project Title: Improving arm function after stroke using task specific training

Human Research Ethics Application (HREA) REFERENCE: 2019/ETH01210

PROTOCOL NUMBER AND DATE: V3 15/09/21

Principal Investigator: Professor Paulette van Vliet, University of Newcastle

---

1. Introduction

You are invited to participate in a research study investigating different training methods for ‘Improving arm function after stroke using task specific training’.

This Participant Information Sheet contains detailed information about the research project. The purpose is to explain to you as openly and clearly as possible the procedures involved in this project before you decide whether or not to take part.

Please read this Participant Information Sheet carefully. Feel free to ask questions about any information in this document. Before deciding whether or not to take part, you might want to talk about it with a relative, friend or your local doctor.

Once you understand what the study is about and if you agree to take part in it, you will be asked to sign a Consent Form. By signing a Consent Form, you indicate that you understand the information and that you give your consent to participate in this research study.

2. Why is this study being done?

This proposal aims to examine the effect of specific exercises on arm function. The study may result in new knowledge to increase understanding of recovery of the arm and hand after stroke that may help to improve future treatments to improve function

3. Why am I being invited to participate in this study?

Because your ability to move your arm has been affected by your stroke. Also because you live within the Hunter New England region of NSW, or the Austin Health area of Melbourne, Victoria. There will be 300 people participating in the study overall.

4. Do I have to take part?

Your participation in this study is voluntary. If you do agree to participate in this research, you are free to change your mind later. If you decide to withdraw from the study, no additional data will be collected from you from this point. Your decision to withdraw will not affect your regular care to which you are otherwise entitled.

You may be taken out of the study, even without your agreement if:

- It is not in your best medical interests to continue
- The study is cancelled.

5. What will happen if I decide to take part?

If you agree to participate in this study, you will receive either a programme of specific exercises for your arm and hand, or continue to receive your usual care. Half of the

participants who enrol in the study will be randomly assigned (like a toss of a coin) to the specific exercises. A computer program will be used to select which participants receive the exercises, and which participants continue to receive their usual care. Each person has an equal chance of being placed in either group.

If you receive the specific exercises, you will receive 14 one hour visits at home from a therapist over a six week period. The exercises will involve doing many repetitions of part of or whole real-life tasks such as reaching to grasp an object. The tasks will be relevant to your everyday life, and will be individualised and varied, using objects of different sizes, weight and shape, in different positions. The therapist will coach you to do the practice, using principles of motor learning. There will also be a short exercise program that you should aim to do every day and you would be asked to keep a diary of when you do them, and how many you do.

If you agree to participate in this study, you will be asked to sign a Consent Form stating that you have read this Information Sheet, you have asked any questions you may have and had these answered to your satisfaction, and that you agree to participate in the study as outlined in this form. We will give you a copy of this Participant Information Sheet to keep.

Following on from your signing the consent form, one of the investigating researchers will:

- Ask you a number of questions in relation to your current condition,
- Perform some assessments to measure your arm and hand function, and the sensation in your hand. These will involve performing a number of different movements, with different objects, and will be performed at home or in a designated clinical room in a hospital or in a research institute which is associated with a hospital by one of the researchers. These assessments will take approximately 2 hours to complete. Evidence of vaccination for Covid19 of research members undertaking assessments will be provided. At the end of the six week period of exercises, or of usual care, these assessments will be performed with you again. They will also be performed a final time at 6 months after you started the exercises, or usual care. If you live within the Hunter New England region of NSW, you may also be asked to visit the movement laboratory at the Hunter Medical Research Institute, at the end of the period of exercises, or of usual care, and at 6 months after you started the exercises, or usual care, so that your arm and hand movements can be recorded and analysed with motion cameras. The visit to the movement laboratory will take approximately one hour.

If you received the specific exercises, you may be invited to take part in one interview, at home or by telephone or video call using zoom via the internet, about your experiences

with the specific training, at the end of the six week period of exercises, and then a second interview 6 months after you started the exercises.

Any information you provide will be kept confidential and coded, so that your name will not appear on any research documents.

The project will start in 2018 and last for four years. The data from all participants will be analysed to determine if the exercises produce any improvement in arm and hand function after stroke. This will be used to inform therapy treatments to improve arm and hand recovery after stroke for different groups of patients. This will prove of great value to future patients.

6. Are there alternatives to participation?

You do not have to participate in this research project. It is entirely your decision. If you decide not to participate, you will still receive your usual care.

7. What are the risks and benefits of taking part?

A program of regular exercise following stroke can have benefits associated with it, including improvement in function and feelings of overall wellbeing. There may be a possibility of some slight soreness from new exercises that a participant may not be used to doing. However, risk from any exercise program can be minimised by adhering to the guidelines and instructions provided to you. If any distress is caused by any of the procedures in the study, including the interview, the following number can be contacted for independent support: StrokeLine 1800787653.

8. Outcome of the study

During the study we will be presenting interim results at various research meetings. No participant will be able to be identified from the results presented. At the end of the study we will analyse the data of all participants together. These data will be published in a

scientific journal.

9. How will I be informed of the results of this research project?

Once the study is complete and the results are known, a written plain English summary of the results of the study will be made available to you upon request. To obtain this please contact the investigator listed in section 15.

10. What will happen to information about me?

Any information obtained in connection with this research project that can identify you will remain confidential and will only be used for the purposes stated in this document. It will only be disclosed with your permission, except as required by law. We plan to publish the results of this study in a scientific journal. In any publication and/or presentation, information will be provided in such a way that you cannot be identified, except with your express written permission.

We will assign each participant a unique code that will be used for all data file names. The information that links your name and contact details to the code will be stored in a locked filing cabinet in a secure room with access limited only to the research team directly involved in this project. The linkage information will be destroyed at the end of the data collection and analysis phase.

Computer files will be stored on password protected computers at the University of Newcastle NSW, La Trobe University in Bundoora Victoria and the Florey Institute of Neuroscience and Mental Health in Parkville Victoria. These computers will be kept in a secure room at each location, with access limited to certain members of the research team.

Information about you may be obtained from your medical record held at health services such as from your local doctor or hospital for the purposes of this research. Any information used from these data sources will be treated completely confidentially.

Information will be kept for a period of fifteen years after the study has been completed.

It is desirable that your local doctor be advised of your decision to participate in this research project. By signing the Consent Form you agree to your local doctor being notified by the project team. Information about your participation in this research project will be recorded in your medical record, with a label bearing the name and duration of the study.

11. How can I access my information?

In accordance with relevant Australian and/or Commonwealth privacy and other relevant laws, you have the right to access the information collected and stored by the researchers about you. You also have the right to request that any information with which you disagree be corrected. Please contact one of the researchers named at the end of this document if you would like to access your information.

12. Is this research project approved?

The ethical aspects of this research project received approval via a National Human Research Ethics Application. This project will be carried out according to the National Statement on Ethical Conduct in Human Research (2007), produced by the National Health and Medical Research Council of Australia. This statement has been developed to protect the interests of people who agree to participate in human research studies.

13. How is this study being paid for?

The study is being funded by a grant from the National Health and Medical Research Council. All of the money being paid from the grant has been deposited into an account managed by the University of Newcastle.

14. Will I be paid for my involvement in this study?

You will not be paid for your participation in this research. However, if you are

required to travel to the movement laboratory, you will receive a $20 Coles/Myer voucher to assist with travel costs.

15. Who can I contact?

The person you may need to contact will depend on the nature of your query. Therefore, please note the following:

<u>For further information:</u>

If you want any further information concerning this project or if you have any medical problems which may be related to your involvement in the project, you can contact

- Principal researcher Professor Paulette van Vliet on 02 4921 7340 or email: paulette.vanvliet@newcastle.edu.au,
- Professor Leeanne Carey, LaTrobe University on 03 94795600 or email: l.carey@latrobe.edu.au
- Dr Meredith Tavener, the Trial Manager on 02 4042 0684 or email: meredith.tavener@newcastle.edu.au

Ethics:

This research has been approved by the Hunter New England Human Research Ethics Committee of Hunter New England Local Health District, Reference 2019/ETH01210.

Complaints about this research:

Should you have concerns about your rights as a participant in this research, or you have a complaint about the manner in which the research is conducted, it may be given to the researcher, or, if an independent person is preferred, please contact the HNE Research Office, Hunter New England Local Health District, Level 3, POD, HMRI, Lot 1 Kookaburra Circuit, New Lambton Heights NSW 2305.

Telephone: 02 4921 4140. Email: HNELHD ResearchOffice@health.nsw.gov.au and quote the reference number: 2019/ETH01210.

Research and Innovation Division

The University of Newcastle

Callaghan NSW 2308

Participant Consent Form

Full Project Title: Improving arm function after stroke using task specific training

Human Research Ethics Application (HREA) REFERENCE: 2019/ETH01210

PROTOCOL NUMBER AND DATE: V3 15/09/21

Principal Investigator: Professor Paulette van Vliet, University of Newcastle

---

Consent Statement -

I have read, or have had read to me in a language that I understand, the Participant Information Sheet and this Consent Form and I understand the purposes, procedures and risks of this research project as described within it.

Pt Initials

☐ I have had an opportunity to ask questions and I am satisfied with the answers I have received.

☐ I freely agree to participate in this research project as described.

☐ I understand that I will be given a signed copy of this document to keep.

Participant's name (printed) ……………………..…………………………………………………
Signature ………………………………………………………….Date ………..…………….
(The participant should print their name, sign and date the form)

Declaration by Recruiting Researcher: I have given a verbal explanation of the research project, its procedures and risks and I believe that the participant has understood that explanation.

Investigator's name (printed) ………………………………………………………………..…..…

Signature of Investigator ……………………………………… Date …………………….

Witness (Required when participant cannot sign for him/herself)

Name of witness (printed) ………………………………………………………………..…..…

Signature of Witness ………………………………………… Date …………………….

Note: All parties signing the consent section must date their own signature.
